# Tsen-Hwang Shaw: Founder of Vertebrate Zoology in China

**DOI:** 10.1007/s13238-020-00780-0

**Published:** 2020-09-04

**Authors:** Fuwen Wei, Dehua Wang

**Affiliations:** 1 Institute of Zoology, Chinese Academy of Sciences, Beijing, 100101 China; 2 University of Chinese Academy of Science, Beijing, 100049 China

**Figure 1:**
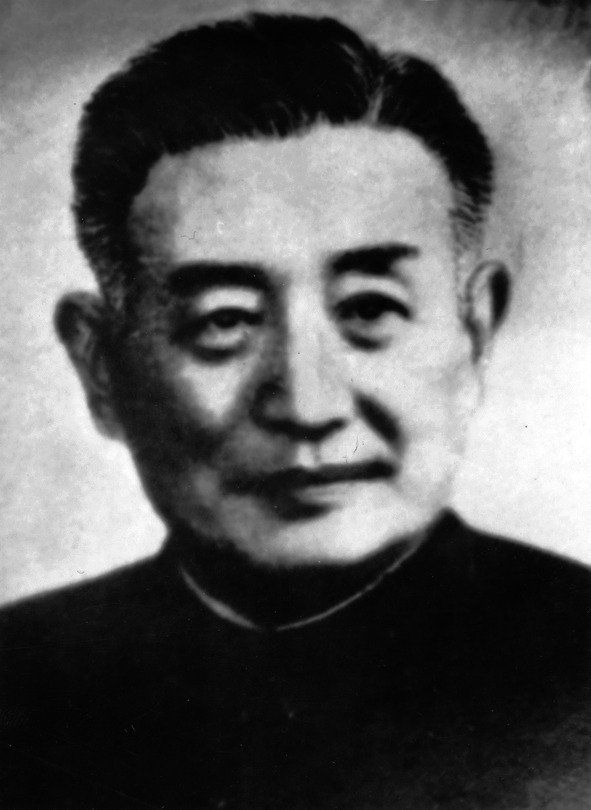
Tsen-Hwang Shaw (1899–1964)

“The founder of vertebrate zoology in China; an erudite scholar of insect, fish, bird and mammal research; combining taxonomy and animal ecology, and pioneering in biostatistics.” These are memorial comments written by Chinese mammalogist Wuping Xia on the 15th anniversary of the death of Professor Tsen-Hwang Shaw (寿振黄, 1899–1964), which offers an overview of the academic contributions of Professor Shaw (Fig. 1).

Professor Shaw was born in Zhejiang Province on February 4, 1899. Growing up in the countryside, he was acutely aware of the constrained circumstances of life in rural areas. To change the backwardness of the countryside in China, he went to National Nanjing Higher Normal School (which later became Southeast University) to study agriculture in 1917. After graduation in 1920, he started to teach biology in a middle school, and he soon realized that biology is the foundation of agriculture and decided to switch from agriculture to biology. In 1921, he was admitted to the Department of Biology at Southeast University at age 22. After receiving a BSc degree in 1925, he went to the United States for further study, and initially enrolled at the University of California, Berkeley and then Stanford University in 1926. He conducted his research on fish taxonomy under the supervision of the famous fish taxonomist D. S. Jordan. In August 1926, Shaw went to Hopkins Marine Station to conduct research on the life history of crustaceans. Later that year, he completed his thesis and obtained a master’s degree. He then returned to the University of California and engaged in research related to ornithology and zoology. In 1927, he successively visited different universities and museums in Philadelphia, Chicago, New York, and Washington to learn the techniques of museum display and taxidermy. The wide range of learning interests and rich research experience in different fields laid a solid foundation for Shaw’s future career.

**Figure 2:**
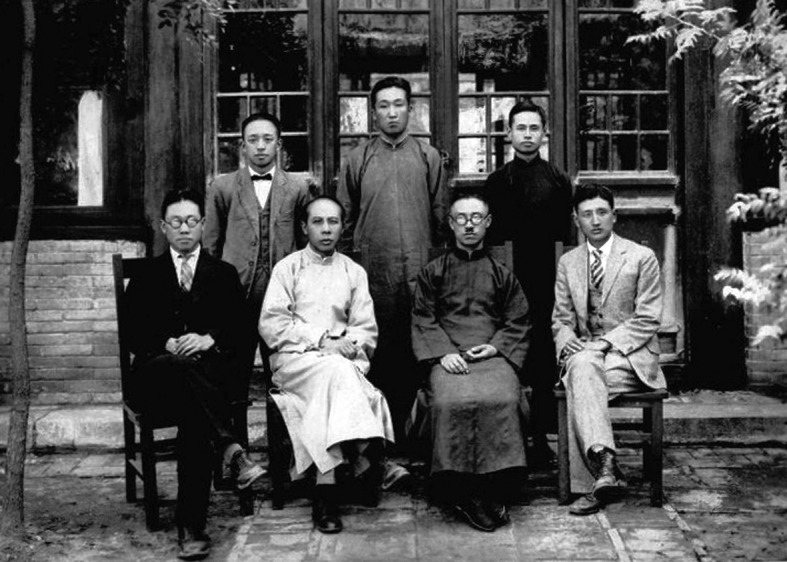
**Photograph at the Establishment of Fan Memorial Institute of Biology in 1928** (Front row, from left: Qi He, Zhi Bing, Xiansu Hu, Tsen-Hwang Shaw (Shou Zhenhuang); Back row, from left: Jiaju Shen, Chengru Feng, Jin Tang)

Professor Shaw returned to China in 1928. He served at Tsinghua University for eight years as a lecturer and then a professor, teaching courses such as comparative anatomy and ichthyology using textbooks written by himself. At the same time, he also worked as a technician of the Animal Department of the Fan Memorial Institute of Biology in Beijing (Fig. 2).

After the founding of the People’s Republic of China, he served as a research fellow and professor in the Committee for Animal Specimens of the Chinese Academy of Sciences (CAS) and Institute of Zoology of CAS. In 1957, he was appointed as a principal investigator and the director of the Mammalogical Research Division of the Institute of Zoology of CAS. In 1959, he served as the director of the Department of Animal Ecology, and in 1962, deputy director of the Institute of Zoology, CAS. During Professor Shaw’s academic career, he published more than 100 papers and related books on vertebrate taxonomy, morphology, paleontology, and ecology.

He was the first Chinese scientist to study ichthyology. Professor Shaw published the book “*Fishes from eastern China*, *with descriptions of new species*” in collaboration with the American ichthyologist B. W. Evermann in 1927 (Evermann and Shaw, 1927, 1927). Later, he continued to carry out groundbreaking studies on the taxonomy, morphology, and ecology of fishes in eastern and northern China, and published a series of research papers (e.g., Shaw, 1929a, b; Shaw and Tchang, 1931; Shaw and Lee, 1939).

He was one of the earliest researchers to study amphibians and reptiles in China. As far back as 1929, Prof. Shaw had investigated the amphibian species of Beiping (currently called Beijing) (Shaw, 1929b) and he also conducted research into the digestive system of turtles in the 1940 (Shaw, 1940).

He was one of the founders of modern ornithology in China. In 1927, Professor Shaw published the paper “A small collection of birds from Fukien” in *Science* (Shaw, 1927), making him the first Chinese scientist to publish an ornithological research paper in this journal. From 1930–1934, he conducted research into the birds of Chefoo (Yantai), Szechwan (Sichuan) and Chekiang (Zhejiang) (Shaw, 1930, 1931, 1932, 1934). In 1936, he published the English-language textbook “*The Birds of Hopei Province* (Two volumes)” (Shaw, 1936), which was the first ornithological book written by a Chinese zoologist with international influence and the first monograph on local fauna organized based on taxonomy. This monograph is regarded as a paradigm of local zoological study, as well as the beginning of vertebrate taxonomy in China.

He was also one of the main promoters of mammalogical research after the founding of People’s Republic of China. To fill in the gap in vertebrate zoology in China, Professor Shaw shifted his research focus to mammals at the age of 65 and established the first mammalogical research division and the first animal ecology laboratory in China. He led many pioneering studies, such as the survey of mammals, and rodent control in Korean pine forestry, and the survey of epidemic hemorrhagic fever in northeastern China. These profound contributions are reflected in a series of works completed in 1958–1962, such as “*A Report on the Mammals of Northeastern China*” (the first regional survey report on mammals), “*Rodent Control in the Direct Seeding of Korean Pine*” (a representative work combining theory and practice), “*Economic Fauna of China*: *Mammals*” (the first monograph on the taxonomy, morphological characteristics, habits, distribution and economic significance of animals nationwide), among other work (e.g., Mammalogical Research Division of the Institute of Zoology, Chinese Academy of Sciences, 1958; Shaw et al. 1958; Shaw, 1962). Through these scientific activities, he cultivated a professional team of mammalogists for the country and had a far-reaching influence on his successors.

He was among the first advocates of wildlife protection and the establishment of nature reserves in China. In 1956, Professor Shaw attended a conference on rodent biology and control in Germany and visited the nature reserves there, which inspired him to propose the establishment of nature reserves to the Yunnan provincial government, together with botanist Cheng-Yih Wu (Zhengyi Wu). Their specific proposals for establishing 24 nature reserves in Yunnan Province were approved and adopted by the government in 1957.

He was a pioneer of research on wildlife hunting in China. After he shifted to mammalogy, he expanded his research field to animal ecology, applied ecology on the basis of taxonomy and zoogeography, and carried out research on the rational utilization of animal resources in the game industry. He was the first to conduct surveys on fur animals in China (Shaw, 1955). He also wrote a manuscript on the biological basis of hunting, and compiled the book “*Illustrated Fur-bearing Mammals in China*” (Shaw, 1958).

Professor Shaw’s research on fish, amphibians, reptiles, birds, and mammals was groundbreaking and made tremendous contributions to the establishment and development of the relevant research areas in China. He also paid much attention and displayed great enthusiasm for educating young mammal experts and animal ecologists, and some of them have become leading scientists in mammalogy and animal ecology in China. Accordingly, he is rightly deemed the founder and pioneer of vertebrate zoology in China.
